# Lupus Antibody Mimicking Reduced Plasmatic Coagulation in a Patient With Atrial Fibrillation and Ischemic Stroke

**DOI:** 10.3389/fneur.2020.00896

**Published:** 2020-08-21

**Authors:** Aydin Huseynov, Verena Haselmann, Maximillian Kittel, Thomas Bertsch, Angelika Alonso, Michael Neumaier, Martin Borggrefe, Ursula Hoffmann

**Affiliations:** ^1^First Department of Medicine, Medical Faculty Mannheim, University of Heidelberg, Mannheim, Germany; ^2^DZHK (Deutsches Zentrum für Herz-Kreislauf-Forschung - German Centre for Cardiovascular Research), Mannheim, Germany; ^3^Medical Faculty Mannheim, Institute for Clinical Chemistry, University of Heidelberg, Mannheim, Germany; ^4^Laboratory Medicine and Transfusion Medicine, Institute of Clinical Chemistry, Nuremberg General Hospital, Paracelsus Medical University, Nuremberg, Germany; ^5^Department of Neurology, Medical Faculty Mannheim, University of Heidelberg, Mannheim, Germany

**Keywords:** lupus anticoagulant, coagulation factor deficiency, thromboplastin, ischemic stroke, anticoagulation

## Abstract

**Background:** Lupus anticoagulant (LA) owns procoagulant properties *in vivo* and prolongs phospholipid-dependent clotting times *in vitro*. The prolonged *in vitro* clotting time can be misinterpreted as a bleeding disorder. In some cases, it is necessary to differentiate LA-associated *in vitro* changes from *in vivo* coagulation factor deficiency. In this case, we used different laboratory testing in a patient with ischemic stroke and reduced prothrombin time (PT) to identify an *in-vitro* effect of LA excluding an *in-vivo* bleeding disorder.

**Methods:** The activity of various coagulation factors was evaluated both with recombinant thromboplastin Innovin (Siemens Healthcare) and reagent tissue extracted thromboplastin Thromborel® (Siemens Healthcare). Moreover, a 1:1 plasma mixing test with standard plasma was performed. In order to exclude the interaction of tromboplastin and LA thromboplastin, an independent global coagulation test, thromboelastography, was used. Diluted-Russel-Viper-Venom (dRVVT) assay was applied to detect the presence of LA detection.

**Results:** The activity of several coagulation factors measured with recombinant thromboplastin Innovin (Siemens Healthcare) showed a reduced activity of the following coagulation factors: Factor V (20.9%), Factor VII (23.8%), Factor X (19.7%) and international normalized ratio (INR) of 2.33. Re-assessment of the factor's activity with another reagent tissue extracted thromboplastin Thromborel® (Siemens Healthcare) showed a normalization of INR and factor's activity in comparison to thromboplastin reagent Innovin®: Factor V (77%), Factor VII (45.4%), Factor X (64.2%), and INR of 1.28. A plasma mixing study with 1:1 standard plasma revealed reduced (<50%) normalization of INR as well as coagulation factor's activity confirming a LA-inhibitor in the patient plasma. Diagnostic LA testing was also performed with dRVVT assay showing a significantly prolonged (112.8 s) test time. Thromboelastography revealed no abnormalities.

**Conclusions:** Different thromboplastin reagents and plasma mixing tests as well as thromboplastin independent coagulation tests may be helpful to differentiate LA and *in vitro* changes from *in vivo* factor deficiency in patients with LA.

## Introduction

Lupus anticoagulant (LA) is an antiphospholipid antibody that binds phospholipids and proteins associated with the cell membrane (1). Phospholipids are essential *in vivo* because of their biological role in cell membranes and are used *in vitro* to accelerate coagulation. LA have procoagulant properties *in vivo*, but since they bind phospholipids in the testing assay, they prolong phospholipid-dependent clotting times *in vitro* (2). The presence of LA and arterial and/or venous thrombosis and/or pregnancy morbidity is one of the characteristics of antiphospholipid syndrome (3). The diagnosis and management of this disease can be challenging, as increased *in vitro* clotting time can be misinterpreted and may delay anticoagulation.

## Case Presentation

A 77-year-old female patient presented to the emergency department with an acute onset of aphasia, dysphagia and right-sided hemiplegia. The patient had a history of hypertension as well as cutaneous lupus erythematosus without any bleeding events. Prior medication was only verapamil (Ca-channel blocker). She did not take any other medication during the last few weeks. The neurological investigation revealed global aphasia, right-sided homonymous hemianopsia, right-sided hemiplegia and hemihypoalgesia. As an acute stroke was suspected, the patient underwent multinodal cranial CT scanning within the first 15 min of arrival in the emergency department to obtain the necessary information needed for further acute treatment.

There were no signs of an acute intracerebral bleeding but a proximal occlusion of the M1 segment of the left middle cerebral artery (MCA) was detected as well as corresponding perfusion deficit in the territory of the left MCA. As the presentation was within 30 min symptom onset, intravenous thrombolysis with a recombinant tissue plasminogen activator, in combination with a mechanical thrombectomy, where a complete recanalization of the MCA could be achieved, was immediately performed. New onset atrial fibrillation, which was detected in the emergency room, was suspected as possible cause of the stroke. The routine blood coagulation tests sampled at admission showed normal platelet count (171/nl) and reduced plasmatic coagulation. International normalized ratio (INR) 2.33 (Reference Range (RR) 0.9–1.15), activated partial thromboplastin time (aPTT) of 30.4 s (RR 15–30 S) (Citrate blood, using reagent Innovin® and Actin FS® on SYSMEX 2100i Siemens Health Care Diagnostics, Marburg, Germany). As no coagulation disorder was known and the test results were completed within 30 min of arrival in the emergency, the thrombolysis therapy was performed.

## Clinical Course After Admission

**Figure 1:**
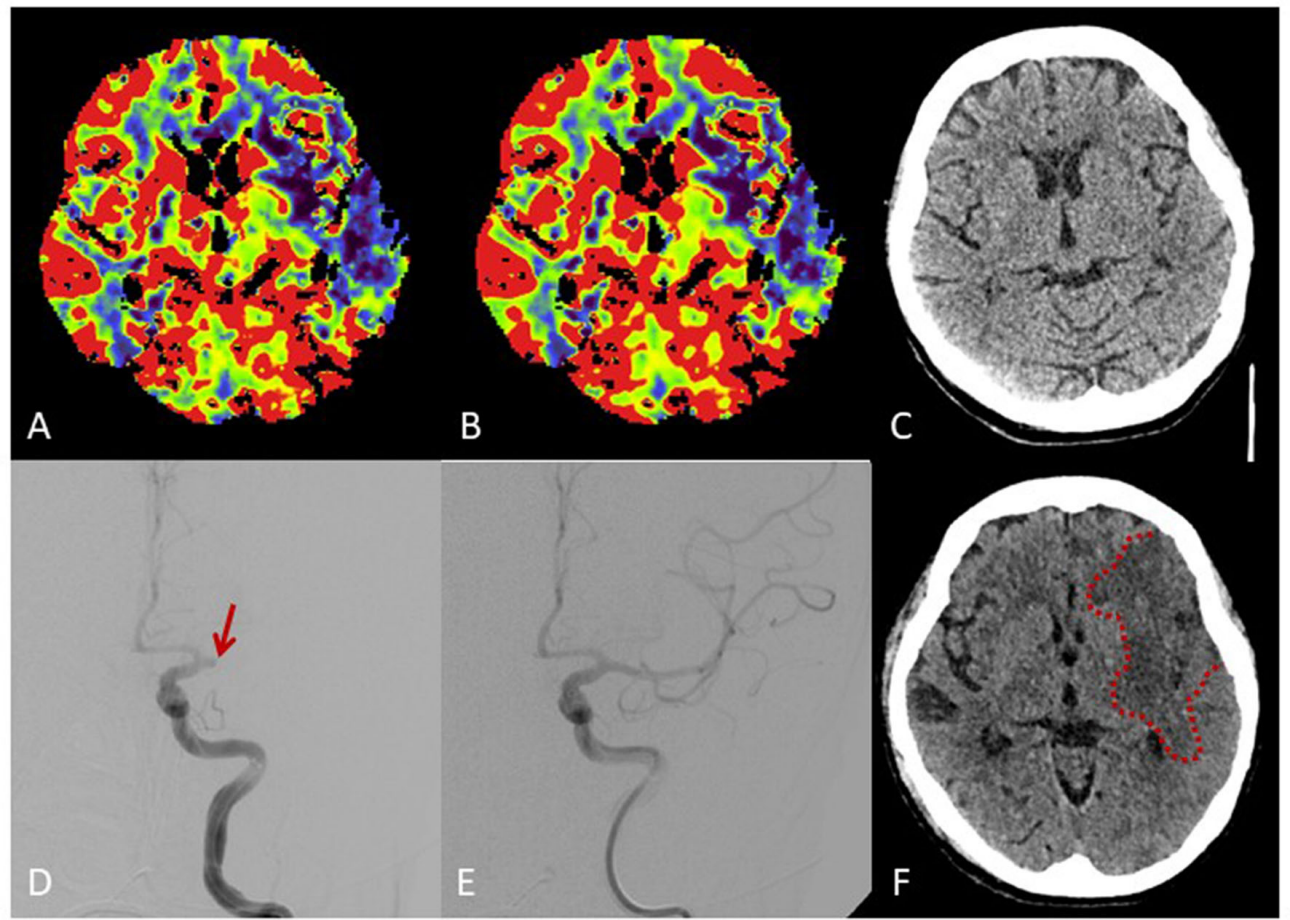
Brain imaging. Multimodal cranial CT imaging at admission shows reduced perfusion in cerebral blood flow maps **(A)** und cerebral blood volume maps **(B)** in the territory of the middle cerebral artery (MCA) without early ischemic changes on non-contrast-CT **(C)**. Angiography **(D)** shows proximal occlusion of the MCA (arrow) with complete recanalization after intravenous thrombolysis and thrombectomy **(E)**. Follow-up CT shows a large frontoparietal MCA infarction despite complete recanalization **(F)**.

The patient was referred to the neurological intensive care unit. A subsequent up cranial CT scanning revealed full demarcation of a large frontoparietal infarction in the territory of the left MCA and no evidence of bleeding despite *in-vitro* reduced plasmatic coagulation (Figure 1).

Because of the repeated reduced plasmatic coagulation test, haemostaseology specialists were involved. Further laboratory investigation and the analysis of the activity of separate coagulation factors revealed a reduced activity of coagulation Factor V (20.9%), Factor VII (23.8%), Factor IX (66%), Factor X (19.7%), Factor XI (53%), Factor XII (26%), and Factor XIII (65%) (Citrate blood, using reagents Innovin®/ActinFS® and SYSMEX 2100i Siemens Health Care Diagnostics, Marburg, Germany. FXIII activity was measured by ammonia released chromogenic assay (Berichrom® F XIII, Siemens Sysmex System CS 5100). Von Willebrand Factor antigen (VWF Ag®, Siemens System BCS®XP) and platelet agglutination caused by ristocetin cofactor assay (INNOVANCE® VWF, Siemens System BCS®XP), fibrinogen (Dade®, Siemens Sysmex System CS 5100), and Factor VIIIc (ActinFS®, Siemens Sysmex System CS 5100) as well as platelet function tests (Platelet Function Assay (PFA-100) und Platelet Aggregation by Born) were normal. The global *in vitro* coagulation test – thromboelastography showed normal test results. The LA test showed pathological results: the diluted-Russel-Viper-Venom (dRVVT) assay was significantly prolonged (112.8 S). The antiphospholipid antibodies were slightly increased in the screening test, however the further IgM and IgG Testing showed results in the RR (Table 1). 1:1 Plasma mixing study with standard plasma revealed a normalization of INR as well as of coagulation factors activity (Table 2) as effect of inhibitory activity of patient plasma. Re-assessment of the factor's activity with another reagent Thromborel® (Siemens Healthcare) showed a normalization of INR and factor's activity in comparison to the previously used thromboplastin reagent Innovin® (Table 2). The results of the test showed the presence of an *in vitro* coagulation factor activity inhibitor having different sensitivity to tissue extracted and recombinant thromboplastin. Furthermore, the thromboplastin independent coagulation test thromboelastography revealed no relevant coagulation disorders (*r*-Time 14.6 min [9–27 Min], *k*-Time 3.2 Min [2–9 min], max. amplitude 60 mm [44–64 mm]). As a haemorrhagic disorder was no longer suspected, the patient was anticoagulated with enoxaparin. During further hospital course, endoscopic gastrostomy was performed because of dysphagia without any bleeding complication and the patient was discharged to a neurological rehabilitation center.

**Table 1:** Initial laboratory parameter.

	**Result**	**Reference range**		**Result**	**Reference range**
Factor II	101%	70–140%	PFA-EPI	117 Sec	8–150 Sec
Factor V	20%	70–120%	Lupus anticoagulant LA1	112.8 Sec	0–44 Sec
Factor VII	23%	70–140%	Lupus anticoagulant LA 2	42.0 Sec	28–32 Sec
Factor VIIIc	188%	70–200%	LA1/LA2 ratio	2.69	0-1,2
Factor IX	66%	70–140%	Lupus sensitive PTT	55.6 Sec	25–34 Sec
Factor X	19%	70–140%	Platelets	167 10E9/L	165-387E9/L
Factor XI	53%	70–140%	Anti-B2-glycoprotein screening	15 U/ml	10 U/ml
Factor XII	20%	70–140%	Anti-B2-glycoprotein IgG	2.50 U/ml	0–8 U/ml
Factor XIII	65%	70–140%	Anti-B2-glycoprotein IgM	3.90 U/ml	0–8 U/ml
INR	2.33	0.9–1.15	Anti-cardiolipin screening	16.30 U/ml	0–10 U/ml
aPTT	30.4	15–30 Sec	Anti-cardiolipin IgG	1.20 U/ml	0–7 U/ml
vWF-Antigen	>200%	70–150%	Anti-cardiolipin IgM	4.50 U/ml	0–10 U/ml
Ristocetin-Cofactor	>150%	70–150%	Anti-Annexin V IgM/IgG	<8 U/ml	0–10 U/ml

*aPTT, activated partial thromboplastin time; INR, international normalized ratio; PFA-EPI, platelet function assay with epinephrine membrane; vWF, von-Willebrand-Factor*.

**Table 2:** Advanced testing of coagulation factor activity.

	**Innovin^®^/ActinFS^®^ for FXII**	**Thromborel^®^**	**50% Patient plasma, 50% standard plasma**	**RR**
INR	2.33	1.28	1.86	0.9–1.15
Factor V	20.9%	77%	33.6%	70–120%
Factor VII	23.8%	45.4%	33.6%	70–140%
Factor X	19.7%	64.2%	37.4%	70–140%
Factor XII	20.9%	–	46.2%	70–140%

*INR, international normalized ratio*.

## Discussion

Both, atrial fibrillation and antiphospholipid syndrome (APS) are associated with cerebrovascular events (4, 5). The state of the art treatment of an acute stroke is thrombolytic therapy with the thrombolytic drug – tissue plasminogen activator alteplase (6). However, impaired coagulation can be a contraindicated for fibrinolytic therapy. Thus, INR values above 1,7 are associated with increased risk of intracranial bleeding. Therefore, thrombolytic therapy is contraindicated in these cases (7). In addition hemorrhagic disorders and a deficiency of coagulation factors, lupus antibodies impair global coagulation tests (8). In the present case, the patient did benefit from thrombolytic therapy and further secondary prevention of thromboembolic events due to APS or atrial fibrillation. However, medical therapy in this case was challenging: on the one hand, there was the need of fibrinolytic therapy and anticoagulation as secondary prevention of thromboembolic events and, on the other hand, *in vitro* signs of hemorrhagic coagulation disorder due to impaired INR and reduced activity of separate coagulation factors. The fact that there were no signs of impaired liver function and no history of bleeding or any hemorrhagic disorders, gave suspicion that the laboratory results do not depict *in-vivo* hemostasis. Furthermore, thrombolytic therapy did not cause any bleeding complications, which could be typical for a case of severe coagulation factor deficiency. Therefore, indirect methods, such as a plasma mixing test and testing with other less lupus sensitive reagents were used.

## Lupus Anticoagulant

LA is one of the most mysterious antibodies in patients with an antiphospholipid syndrome. On the one hand, an antiphospholipid antibody may cause a phospholipid-dependent prolongation of the clotting time *in vitro* and, on the other hand, it is associated with an increased risk of thrombosis (9). This ambivalence is based on the feature of building complexes which slow down coagulation reactions *in vitro* by forming stable complexes on coagulation active phospholipids (8). Laboratory diagnostic tests for the LA are heterogeneous and include phospholipid-dependent clotting assays, plasma mixing studies, and demonstration of the phospholipid-dependency of the inhibitory activity. Due to the heterogeneity of antibodies, reagents and the variability of the analyser, the general algorithms of LA detection are missing (3).

LA is usually detected with clotting assays based on aPTT and dRVVT. Both can be performed at low and high phospholipid concentration, or on 1:1 mixtures of tested sample and a normal plasma pool (10). Some prothrombin assays have demonstrated to be sensitive to the effects of LA, leading to a prolonged PT and consequently false INR values. Thromboplastin reagents are widely used as clotting activator *in vitro* and they contain either recombinant tissue factor or tissue factor extracted from the brain and placental sources. Recombinant thromboplastins have been discussed to be more sensitive to variation in the presence of LA. Accordingly, tissue extracted thromboplastins are more suitable for measurements (11). The reason for such correlations is not known. Possible explanation is an interaction of LA either with recombinant replicated tissue factor or a different phospholipid composition (12). In summary, the fact of different results using different reagents could be a good evidence of the existence of LA in the present case. Both, plasma mixing test and the use of different reagents were helpful to choose the anticoagulation strategy for this patient. Furthermore, thromboplastin independent coagulation tests, such as thromboelastography, should be used to rule out a severe bleeding coagulation disorder.

## Data Availability Statement

The original contributions presented in the study are included in the article/supplementary material, further inquiries can be directed to the corresponding author/s.

## Ethics Statement

Written informed consent was obtained from the individual(s), and minor(s)' legal guardian/next of kin, for the publication of any potentially identifiable images or data included in this article.

## Author Contributions

AH, TB, MB, and UH contributed to the conception and design of the paper. VH and MK contributed to preparing laboratory data. MN and AA contributed to developing the idea of the study and revising the article critically for important intellectual content. All authors contributed to the article and approved the submitted version.

## Conflict of Interest

The authors declare that the research was conducted in the absence of any commercial or financial relationships that could be construed as a potential conflict of interest.

## Abbreviations

aPTT: activated partial thromboplastin time
APS: antiphospholipid syndrome
dRVVT: diluted-Russel-Viper-Venom
INR: international normalized ratio
LA: lupus anticoagulant
MCA: middle cerebral artery.
